# Limitations in Activities of Daily Living among Older Adults with COPD, Asthma, or Asthma-COPD Overlap Residing in Spain

**DOI:** 10.3390/ijerph20043467

**Published:** 2023-02-16

**Authors:** Sheila Sánchez Castillo, Lee Smith, Arturo Díaz Suárez, Guillermo Felipe López Sánchez

**Affiliations:** 1 Faculty of Sports Sciences, University of Murcia, 30720 San Javier, Spain; 2 Centre for Health Performance and Wellbeing, Anglia Ruskin University, Cambridge CB1 1PT, UK; 3 Division of Preventive Medicine and Public Health, Department of Public Health Sciences, School of Medicine, University of Murcia, 30120 Murcia, Spain

**Keywords:** lung diseases, elderly, functional status, healthy ageing

## Abstract

Activities of daily living (ADL) may be limited by the presence of chronic diseases, and limitations in ADL contribute to an increased risk of falling. In people with asthma, chronic obstructive pulmonary disease (COPD), and asthma–COPD overlap (ACO), ADL may be affected owing to poor asthma control and COPD ventilatory limitations. The aim of this study was to establish the differing prevalence of limitations in ADL among older Spanish adults with chronic respiratory diseases (COPD, asthma, and ACO). Data from the Spanish National Health Survey were analyzed. The sample was composed of 944 older adults aged ≥65 years and with a positive diagnosis of COPD (*n* = 502), asthma (*n* = 241), or ACO (*n* = 201). Five basic activities of daily living (BADL) and seven instrumental activities of daily living (IADL) were studied. Frequency and percentages were used to describe sample characteristics and limitations of ADL. Significant differences were analyzed using chi-square tests. Results revealed a significant higher number of older adults with COPD (34.8%) and asthma (32.5%) without limitations in doing hard housework in comparison to ACO (17.8%). Regarding meal preparation, a higher percentage of asthmatics without difficulties (77.7%) and a lower percentage with many difficulties (2.6%) were observed in comparison to ACO (64.8%–10.2%). No differences were found in BADL, with approximately 80–90% without limitations. It seems that limitations in IADL vary according to the type of chronic pulmonary diseases, but further research is needed to clarify why differences were found only for preparing meals and hard housework. These findings should be considered in the design of interventions to promote ADL in older adults with respiratory disease.

## 1. Introduction

Chronic respiratory diseases are important public health problems owing to a high prevalence and associated morbimortality. Both chronic obstructive pulmonary disease (COPD) and asthma are two of the “big five” lung diseases, according to the Forum of International Respiratory Societies [1]. Although onset of asthma is very common among children, data from the 2019 Global Burden of Disease found a worldwide prevalence of asthma among those aged 65–89 years: between 4.5–6.5% [2]. Moreover, mortality rates for asthma are higher among older adults, which may be related to an insufficient diagnosis and delayed treatment [3]. As for COPD, among the same age group, a prevalence between 14.4% and 17.0% was found [2], and it was the third leading cause of death in 2019 [4].

These diseases are predominantly characterized by respiratory symptoms including dyspnea, cough, wheezing, chest tightness, and airflow limitation but with some differences between each disease. Mainly, asthma symptoms and airflow limitation vary in frequency and intensity spontaneously or with bronchodilators, while in COPD, they are persistent, and patients may have a history of exposure to toxic particles, low birth weight, or respiratory illness such as tuberculosis [5,6]. Moreover, some people have persistent airflow limitation together with clinical features of both diseases, for which the term asthma–COPD overlap (ACO) is used. This designation does not mean a new single disease but is a word coined by the Global Initiative for Asthma to describe the combination of both diagnoses of asthma and COPD [6]. Importantly, all those characteristics consequently deteriorate health status.

Health status refers to an individual’s level of wellness and illness considering their biological or physiological dysfunction, symptoms, and functional impairment [7]. It may include two components: health-related quality of life and functional status. Functional status describes the ability to perform activities of daily living (ADL) [8]. These activities are tasks of everyday life. The basic activities of daily living (BADL) include essential activities necessary for an independent daily life such as eating, bathing or showering, dressing, getting into or out of a bed or chair, and getting around inside the home [9]. The instrumental activities of daily living (IADL) are more complex activities than BADL in terms of cognitive and motor level, and they are related to independent living and include activities such as preparing meals, managing money, shopping, doing housework, and using a telephone [10]. The IADL can also be divided into physically demanding household activities such as preparing meals, shopping, and light-hard housework and cognitive IADL, such as managing money or using the telephone [11,12,13]. Functional impairment in ADL can be owing to several factors, including, for example, increased age, daily alcohol consumption, cognitive impairment, the onset of depression, and increasing chronic conditions [14]. ADL can be limited by the presence of chronic diseases [15], including COPD and asthma. Moreover, limitations in BADL and IADL are significantly associated with higher fall risk in older adults, as people with limitations in BADL and IADL usually present poor balance, slow reaction speed and lower extremity weakness, which are primary contributors to higher fall risk in older adults [16].

Previous studies reported higher ADL impartment in those with asthma compared to those without [3,17]. Importantly, poor asthma control increases the probability to have limitations in ADL among asthmatic older adults [18,19]. It is known that physical activity (PA) can help in asthma control [20,21], and practicing it may consequently reduce those limitations. Similarly, ventilatory limitations suffered by COPD patients, such as absence of ventilatory reserve, decrease of inspiratory reserve volume, and dynamic hyperinflation during ADL, are responsible for poorer ADL outcomes and higher complaints of dyspnea while performing activities [22]. Cognitive impairment associated to COPD could also affect ADL [23,24], but there is no clear consensus as to whether it predisposes people with COPD to impairment in ADL [25]. However, COPD patients can increase the level of independence needed to perform ADL by improving functional exercise capacity, reducing symptoms, and increasing expiratory muscle strength [26]. The use of simple energy conservation techniques can also be useful to avoid dynamic hyperinflation when performing ADL [27].

To the best of the author’s knowledge, previous literature analyzing differences in ADL limitations between people with asthma, COPD, and ACO is scarce. Only one paper exists on this topic, showing that level of dyspnea during personal care and leisure ADL recorded using the London Chest ADL scale was significantly higher in people with COPD than asthmatics [28]. Given this background and the importance of independence for a better quality of life among older people with chronic respiratory diseases, the main aim of this study was to establish the differing prevalence of limitations in BADL and IADL among older adults residing in Spain with chronic respiratory diseases (COPD, asthma, and ACO). It is hypothesized that limitations may be higher in people with COPD and ACO than asthmatics, owing to the permanent airflow limitations, specifically in activities that require higher physical implications.

## 2. Materials and Methods

### 2.1. Study Design

The present study is a cross-sectional investigation that analyses data from the Spanish National Health Survey 2017, which was carried out in Spain between October 2016 and October 2017. The present paper follows the STROBE (STrengthening the Reporting of OBservational studies in Epidemiology) checklist.

Methodological details have previously been published [29]. In brief, a three-stage sampling method was used to select participants. Firstly, census sections were considered. Secondly, a systematic sampling defined the family dwellings, and finally, a random Kish method was applied to select an adult (≥15 years) from each dwelling. Data were collected in the participant’s dwelling by using a computer-assisted personal interviewing method.

The present investigation was performed in compliance with Declaration of Helsinki of the World Medical Association. The present analyses were approved by the Ethical Research Committee of the University of Murcia.

### 2.2. Sample

A total of 23,089 people residing in Spain completed the survey. Inclusion criteria were: (1) being at least 65 years old, (2) having a positive diagnosis of chronic respiratory diseases (COPD, asthma, or ACO). People were considered to have positive diagnosis of COPD/asthma/ACO if they answered affirmatively to the question: have you been told by a doctor that you suffer from COPD/asthma/COPD and asthma? People younger than 65 years were excluded because they did not answer the questions about limitations in BADL and IADL. Finally, a total of 944 older adults with COPD (*n* = 502), asthma (*n* = 241), or ACO (*n* = 201) residing in Spain were included in these analyses.

### 2.3. Variables

#### 2.3.1. Sociodemographic Characteristics

Sociodemographic, behavioral, and anthropometric variables were used to characterize the sample and to study the differences between people with COPD, asthma, and ACO. All data were treated as categorical variables except age, which was treated as a continuous variable to characterize the whole sample, and the number of concurrent comorbidities. Sex was a dichotomic variable (men, women). Age was classified into three groups based on previous literature [30,31,32]: 65–74 years old, 75–84 years old, and older than 84 years old. Marital status was treated as a dichotomic variable: married vs. not married. Those single, separated, divorced, and widowed were included in the not married group. Cohabiting was also divided in two categories: yes if they were living in couple and no if they were not. Education was classified in levels A, B, or C, following the Spanish Classification of Education [33]. Level A included people with a level equal or lower than the first period of secondary, level B included people with second period of secondary and post-obligatory studies (not tertiary), and level C were those with tertiary education. Harmful substance consumption was categorized into three and four categories, respectively: current, ex, and never smoker and daily, weekly, monthly, or no alcohol consumption in the past 12 months. Body mass index (BMI) was calculated as weight (kilograms) divided by height (meters) squared and classified as normal weight (18.5–24.9 kg/m^2^), overweight (25–29.9 kg/m^2^), and obesity (≥30 kg/m^2^) [29]. The presence of comorbidities was classified as positive if they had at least one disease in addition to COPD and/or asthma and negative if they had no other chronic diseases.

#### 2.3.2. Dependent Variables

The dependent variables analyzed were the limitations in ADL included in the Spanish National Health Survey 2017. They are the same that are used in the European Health Interview Survey and were selected according to the International Classification of Functioning, Disability, and Health [34]. Those variables measure the difficulties that older adults found when doing basic and instrumental activities. BADL included the following variables:

- Feeding: difficulties in taking food from the plate and bringing it to mouth, to bring a glass to mouth, to cut the food, to use a fork and a spoon, spread jam or butter on a slice of bread, etc.;
- Sitting up, getting up, and laying down: difficulties in doing those actions without any help;
- Dress and undress: difficulties in taking clothes from the wardrobe, putting them on, buttoning them, and tying the shoes;
- Toilet: difficulties using toilet paper, wiping themselves, and taking clothes off before and after using the toilet;
- Shower: difficulties washing and drying the whole body and getting in and out of the shower or bathtub.

IADL included the following variables:

- Preparing their own meals: difficulties preparing their own meals;
- Phone: difficulties making and answering calls;
- Shopping: difficulties with going shopping;
- Medication: difficulties taking their medication in the correct dose;
- Light housework: difficulties doing activities such as cooking, washing dishes, ironing, and taking care of children;
- Hard housework: difficulties carrying heavy shopping, cleaning windows, or cleaning the floor with a brush;
- Money management: difficulties, for example, with paying their own bills.

All variables were classified in four groups according to different difficulty levels: (1) no difficulty if they have no limitations in performing the activity; (2) some difficulty; (3) a great deal of difficulty; and (4) Cannot complete the task if they cannot perform the activity without assistance.

#### 2.3.3. Independent Variable

The variable diagnosis of chronic respiratory diseases was categorized into three groups: COPD if they had a positive diagnosis of COPD, asthma if they had a positive diagnosis of asthma, and ACO if they had positive diagnoses of both asthma and COPD.

### 2.4. Statistical Analysis

Frequency and percentages were used to describe sample characteristics and limitations of ADL in the whole sample and according to respiratory chronic disease. All variables analyzed were categorical, then significance differences were analyzed using chi-square tests. For pairwise comparisons, custom tables with pairwise Z-tests output were used.

Multinomial logistic regression analyses were also carried out to identify the probability of having some limitations, many limitations, or not being able to do the activity without help considering the respiratory disease type. The reference category for the dependent variables was having no limitations in performing the activity. For the independent variable, the reference category was having ACO. The models were adjusted for age, sex, marital status, cohabiting, education level, smoking habits, alcohol consumption, and BMI.

Statistical analyses were carried out using the Statistical Package for Social Sciences v.26. Statistical significance was set at *p* < 0.05.

## 3. Results

A total of 944 Spanish residents aged 65 years and over (55% women) had a positive diagnosis of chronic respiratory diseases, COPD (53.2%), asthma (25.5%), and ACO (21.3%). The mean age of the sample was 76.9 (±7.5) years old, with an age range between 65 and 100 years. In terms of education, 84.1% of the sample had not finished secondary education. Approximately half of the sample were married (51.1%) and living together (50.3%). Regarding harmful substance consumption, 45% were current and ex-smokers, and 58.3% were alcohol consumers, with 24.1% being daily drinkers. In terms of physical characteristics, 75.2% were overweight and obese. Considering other diseases, 98.3% had at least one comorbidity, with a mean of 6.76 concurrent comorbidities. Specifically, sample characteristics according to positive diagnoses of COPD, asthma, or ACO are shown in Table 1. Those with BMI lower than 18.5 kg/m^2^ were excluded from the sample characteristics analyses, as there were no cases with asthma and ACO, and there were only eight cases with COPD. Finally, owing to people that did not answer to questions regarding height and/or weight, a total of 9.4% cases were lost from BMI.

Table 1 shows significant differences according to sex, marital status, living together, smoking, and alcohol. The percentage of men with COPD was significantly higher than asthma or ACO, while in women, it was significantly lower. Similarly, the percentage of older adults with COPD who were married or living together was higher than asthmatics or ACO. In those not married or not living as a couple, frequencies were higher among asthmatics and ACO in comparison to those with COPD. In relation to harmful substances, the percentage of current and ex-smokers was significantly higher among people with COPD in comparison to asthmatics. Moreover, the percentage of non-smokers was higher among asthmatics and ACO in comparison to COPD, but when comparing only asthmatics and ACO, the frequency of non-smokers was significantly higher among those with asthma. Daily alcohol consumption was significantly higher in people with COPD in comparison to asthmatics, and no alcohol intake was higher among asthmatics in comparison to those with COPD.

Table 2 describes the limitations in performing ADL by older adults with respiratory diseases in general and with COPD, asthma, or ACO in particular, analyzing significant differences between the three (COPD-asthma-ACO).

Table 2 indicates that there were no significant differences in limitations in performing BADL independent of the respiratory disease. In general, there was a higher percentage of older adults not having any limitations in feeding (92.1%), sitting up, and standing up (78.6%), dressing (75.6%), going to the toilet (83.8%), and having a shower on their own (71.7). The most difficult activity for Spanish people with COPD, asthma, or ACO was having a shower, with a 11%, 9.5%, and 11.9%, respectively, who cannot do it alone. On average, 80.4% had no limitations in carrying out BADL.

Table 3 shows the limitations in performing IADL for older adults with respiratory diseases in general and analyzes the differences according to the three different diagnoses: COPD, asthma, and ACO. Those who did not answer questions and those who answered that they did not try or need to do them were excluded from that specific analysis. The percentage of older adults excluded was 12.3% for the variable of limitations in preparing their own meals, 1.6% for telephone use, 5.0% for shopping, 1.6% for medication, 10.2% for light housework, 9.0% for hard housework, and 3.2% for the limitations in money management.

Table 3 shows higher percentages of older adults without limitations in preparing their own meals, using the phone, taking their medication, and managing their money. Around half of the sample had some/many of limitations or were not able to go shopping and do light housework. The percentage of older adults who were limited or could not execute hard housework was 69.2%. When analyzing differences between COPD, asthma, and ACO diagnoses, only preparing their own meals and hard housework revealed significant differences. Having no limitations in preparing meals was more common among asthmatics in comparison to those with ACO. Similarly, having many limitations was more common in those with ACO. Regarding hard housework, the frequencies of older adults without difficulties were significantly higher among those with COPD and asthma.

For the variables phone, shopping, medication, and money, significant differences between COPD, asthma, and ACO were not found. However, for all these variables, the percentage of the sample without limitations was higher among asthmatics. On average, 66.8% had no limitations in IADL.

Multinomial logistic regression revealed significant higher probability among those with COPD for presenting some limitations for dressing and undressing (OR: 2.028 [1.081–3.772]; *p* = 0.028), toileting (OR: 2.224 [0.999–4.951]; *p* = 0.050), and showering (OR: 2.227 [1.155–4.294]; *p* = 0.017) and for not being able to dress and undress without help (OR: 3.319 [1.286–8.569]; *p* = 0.013). Regarding IADL, positive correlations were also found among those with COPD for not being able to prepare their own meal without help (OR: 1.938 [1.011–3.717]; *p* = 0.046). On the contrary, a lower probability of having some limitations (OR: 0.467 [0.260–0.838]; *p* = 0.011) and many limitations (OR: 0.546 [0.299–0.999]; *p* = 0.050) in doing hard housework activities was significant among those with COPD. In asthmatics, a lower probability of having some limitations in preparing their own meals (OR: 0.238 [0.080–0.706]; *p* = 0.010) and doing light housework (OR: 0.436 [0.242–0.787]; *p* = 0.006) and hard housework activities (OR: 0.389 [0.205–0.741]; *p* = 0.004) was found as well as for having many limitations (OR: 0.360 [0.184–0.705]; *p* = 0.003) and not being able to do hard housework alone (OR: 0.411 [0.217–0.778]; *p* = 0.006).

## 4. Discussion

Our main findings reveal that there were no differences in terms of limitations in BADL between older adults residing in Spain with COPD, asthma, and ACO; approximately nine out of ten had no limitations for feeding, 84% for toilet activities, and 77% for sitting and getting up and dressing. Showering was the most limited activity, with approximately 18% who could not do it on their own or could do it but with great difficulties. On the contrary, regarding IADL, the number of people with ACO with many difficulties in preparing their own meals was significantly higher, and the number of those with no limitations was lower in comparison to asthmatics. The number of older adults with ACO with no limitations in doing hard housework was also lower in comparison to those with only COPD or asthma. To the best of the authors’ knowledge, there is no previous literature that has analyzed differences in ADL limitations between people with COPD, asthma, and ACO. It is elusive as to why significant differences only appear in activities such as preparing their own meals or doing hard housework, so future research that objectively tests physical function is now required to elucidate potential mechanisms.

Previous systematic reviews and meta-analysis [35,36] have found that older adults who are more physically active have a reduced level of disability. A recent study that established PA levels in Spanish adults with chronic respiratory diseases revealed a higher percentage of participants categorized as having a low PA level among older adults with ACO (45.5%) in comparison to COPD (42.1%) and asthma (38.7%) [37,38,39]. Indeed, low levels of PA could be related with the higher limitations found in activities such as preparing their own meals and hard housework, which may need a higher level of physical fitness to perform.

The present results also revealed that activities with higher cognitive implications, such as phone use and money management, were not significantly different between people with COPD, asthma, and ACO, with a higher percentage of older adults without limitations in both activities (79.8% for money and 81.4% for phone). These findings suggest that differences previously found in cognitive impairment between COPD and asthma [40] may not be strong enough to influence BADL and IADL.

When considering only COPD patients, Annegarn et al. revealed that mobility activities such as walking, climbing stairs, and cycling were the most limited, followed by showering, gardening, cleaning the floor, and dressing/undressing [41]. The present study also showed greater limitations in showering than dressing, but the other activities were not analyzed in this study. Recently, Kaptain et al. analyzed 47 different ADL, both BADL and IADL, among Danish adults with COPD aged 46–87 years [42]. The main findings reported that the most affected BADL were moving within or outside the home and dressing, bathing, and pedicuring; and the most affected IADL were weekly cleaning, washing by hand, going by train, and weekly shopping. The most competently completed BADL were calling for attention, washing hands/face/teeth, feeding activities, toileting, and using the telephone; and the most competent IADL were preparing cold meals and heating up food. The most competent BADLs concur with our results for people with COPD, with more than 90% being competent in feeding, 82.3% in toileting, and 80.1% in telephone use. Regarding IADL, the present results showed that the most limited activities for people with COPD were those related to housework, which almost matches the findings of Kaptain et al. However, due to the differences in the analyzed IADL and age of the sample, this comparison should be considered under its limitations.

Woods et al. analyzed limitations in six BADL among American asthmatics and found that walking was the most limited activity, with 26.4% of the overall sample having limitations (unable to do it or only with many difficulties), followed by transferring in and out of the bed or chair (11.3%), toileting (5.8%), bathing (5.5%), dressing (5.1%), and eating (2.7%). Moreover, this study also found reduced limitations among those asthmatics with good asthma control for all the activities except for eating [18]. In comparison to our results in Spanish asthmatics, there are some differences, with showering proving the most limited activity, with 13.6% of older adults having many difficulties or being unable to do it without help. Dressing was limited in 9.6% of asthmatics, followed by toileting (7.1%) and sitting and getting up (6.6%). Both studies concur that eating is the least limited activity.

In relation to people with ACO, Fragoso et al. [43] found higher odds of having prevalent disability in BADL (bathing, dressing, or getting around the house) among American adults with ACO aged 40–85 (OR: 1.91 [1.12–3.25]) in comparison to those with asthma alone. On the contrary, the present study did not show significant differences in BADL between people with asthma and ACO. This may be due to differences in sample characteristics such as age. Regarding harmful substances consumption, the present study reveals a healthier lifestyle among asthmatics, showing higher percentages of non-smokers and lower frequencies of daily alcohol consumers than in people with COPD or ACO. Both smoking and alcohol consumption are important PA correlates. Azagba and Sharaf concluded that levels of PA in older adults who smoke were lower in comparison to non-smokers [44]. However, the results of the present study did not show higher limitations in COPD or ACO in comparison to asthmatics in relation to housework activities, which may require better physical fitness. In relation to alcohol, previous investigations suggest a positive relationship between alcohol consumption and PA across all ages [45,46]. Indeed, a recent study revealed that older adults with daily alcohol consumption were associated with higher PA levels. [47] Then, higher limitations in hard housework among people with ACO could not be explained because of a reduced alcohol intake since daily alcohol consumption was higher in those with ACO (23.9%) than in asthmatics (17.8%).

Results from the present study should be considered in light of their limitations. First, this study had a cross-sectional design, so causality cannot be established. People were not excluded if they were suffering from another disease apart from COPD and/or asthma, and that could introduce bias in the analysis. However, comorbidities are very common among older adults. Additionally, the present results did not explore the differences with their age-matched individuals without respiratory diseases. Given that there were few differences found in ADL limitations between these three diagnoses, future research should analyze differences within their pairs without chronic respiratory diseases.

## 5. Conclusions

In conclusion, there were no significant differences between older Spanish adults with COPD, asthma, and ACO in their limitations in performing BADL. Considering IADL, only preparing their own meals was found to be more difficult for people with ACO than for asthmatics, and a lower proportion of the sample with ACO could do hard housework without limitations in comparison to those with only COPD or asthma. However, around seven out of ten had no limitations in carrying out ADL. These findings, together with a complete assessment to identify individual limitations, should be considered in the design of interventions to promote ADL in older adults with COPD, asthma, or ACO.

## Figures and Tables

**Table 1:** Differences in sample characteristics considering chronic respiratory diseases diagnosis.

		*n*	COPD ^a^ (*n* = 502)	Asthma ^b^ (*n* = 241)	ACO ^c^ (*n* = 201)	*p*
Sex	Men	425	279 (55.6) ^b,c^	73 (30.3)	73 (36.3)	**<0.001**
	Women	519	223 (44.4)	168 (69.7) ^a^	128 (63.7) ^a^	
Age	65–74	394	213 (42.4)	94 (39.0)	87 (43.3)	0.170
	75–84	390	197 (39.2)	115 (47.7)	78 (38.8)	
	+85	160	92 (18.3)	32 (13.3)	36 (17.9)	
Marital status	Married	483	292 (58.2) ^b,c^	102 (42.3)	89 (44.3)	**<0.001**
	Not married	461	210 (41.8)	139 (57.7) ^a^	112 (55.7) ^a^	
Living together	Yes	475	290 (57.8) ^b,c^	101 (41.9)	84 (41.8)	**<0.001**
	No	469	212 (42.2)	140 (58.1) ^a^	117 (58.2) ^a^	
Education	Level A	795	405 (80.7)	211 (87.6)	179 (89.1) ^a^	**0.020**
	Level B	96	48 (9.6)	11 (4.6)	10 (5.0)	
	Level C	80	49 (9.8)	19 (7.9)	12 (6.0)	
Smoking	Current	80	55 (11.0) ^b^	9 (3.7)	16 (8.0)	**<0.001**
	Ex	345	213 (42.4) ^b^	61 (25.3)	71 (35.3)	
	No	519	234 (46.6)	171 (71.0) ^a,c^	114 (56.7) ^a^	
Alcohol	Daily	229	138 (27.5) ^b^	43 (17.8)	48 (23.9)	**0.005**
	Weekly	70	47 (9.4)	11 (4.6)	12 (6.0)	
	Monthly	157	81 (16.1)	47 (19.5)	29 (14.4)	
	No	488	236 (47.0)	140 (58.1) ^a^	112 (55.7)	
BMI	NW	212	122 (26.5)	47 (21.6)	43 (23.4)	0.156
	OW	357	195 (42.3)	93 (42.7)	69 (37.5)	
	OB	286	136 (29.5)	78 (35.8)	72 (39.1)	
	Lost	89	41	23	17	

COPD, chronic obstructive pulmonary disease; ACO, asthma–COPD overlap; Level A, ≤1st period secondary; Level B, 2nd period secondary and post secondary (not tertiary); Level C, tertiary; BMI, body mass index; NW, normal weight (18.5–24.9 kg/m^2^); OW, overweight (25–29.9 kg/m^2^); OB, obesity (≥30 kg/m^2^). Chi-square test. Statistical significance: ***p* < 0.05**. Superscripts ^a,b,c^ indicate significant differences in pairwise comparisons after Bonferroni correction. ^a^ Significant differences with COPD; ^b^ significant differences with asthma; ^c^ significant differences with ACO.

**Table 2:** Limitations to perform Basic Activities of Daily Living (BADL) in older adults with COPD, Asthma or ACO.

		Resp. Dis.	COPD	Asthma	ACO	*p*
Feeding	No	869 (92.1)	455 (90.6)	226 (93.8)	188 (93.5)	0.660
	Some	39 (4.1)	23 (4.6)	9 (3.7)	7 (3.5)	
	Many	18 (1.9)	13 (2.6)	2 (0.8)	3 (1.5)	
	Cannot do it	18 (1.9)	11 (2.2)	4 (1.7)	3 (1.5)	
Sitting and getting up	No	740 (78.6)	387 (77.1)	199(82.6)	154 (76.6)	0.253
	Some	102 (10.8)	54 (10.8)	26 (10.8)	22 (10.9)	
	Many	58 (6.1)	33 (6.6)	8 (3.3)	17 (8.5)	
	Cannot do it	44 (4.7)	28 (5.6)	8 (3.3)	8 (4.0)	
Dressing	No	714 (75.6)	369 (73.5)	194 (80.5)	151 (75.1)	0.110
	Some	110 (11.7)	64 (12.7)	24 (10.0)	22 (10.9)	
	Many	56 (5.9)	27 (5.4)	11 (4.6)	18 (9.0)	
	Cannot do it	64 (6.8)	42 (8.4)	12 (5.0)	10 (5.0)	
Toilet	No	791 (83.8)	413 (82.3)	210 (87.1)	168 (83.6)	0.116
	Some	64 (6.8)	37 (7.4)	14(5.8)	13 (6.5)	
	Many	36 (3.8)	19 (3.8)	4 (1.7)	13 (6.5)	
	Cannot do it	53 (5.6)	33 (6.6)	13 (5.4)	7 (3.5)	
Shower	No	677 (71.7)	354 (70.5)	183 (75.9)	140 (69.7)	0.122
	Some	101 (10.7)	60 (12.0)	25 (10.4)	16 (8.0)	
	Many	64 (6.8)	33 (6.6)	10 (4.1)	21 (10.4)	
	Cannot do it	102 (10.8)	55 (11.0)	23 (9.5)	24 (11.9)	

Resp. Dis., respiratory disease (COPD, asthma, ACO); COPD, chronic obstructive pulmonary disease; ACO, asthma–COPD overlap. No, if they have no limitations to perform the activity; Some, if they have some difficulties; Many, if they have many difficulties; Cannot do it, if they cannot perform the activity without help. Chi-square test. Statistical significance: ***p* < 0.05**.

**Table 3:** Limitations in performing Instrumental Activities of Daily Living (IADL) in older adults with COPD, asthma, or ACO.

		Resp. Dis.	COPD ^a^	Asthma ^b^	ACO ^c^	*p*
Preparing their own meals	No	600 (72.5)	308 (72.8)	178 (77.7) ^c^	114 (64.8)	**0.019**
	Some	87 (10.5)	40 (9.5)	23 (10.0)	24 (13.6)	
	Many	48 (5.8)	24 (5.7)	6 (2.6)	18 (10.2) ^b^	
	Cannot do it	93 (11.2)	51 (12.1)	22 (9.6)	20 (11.4)	
Phone	No	756 (81.4)	395 (80.1)	205 (85.8)	156 (79.2)	0.538
	Some	73 (7.9)	41 (8.3)	15 (6.3)	17 (8.6)	
	Many	41 (4.4)	24 (4.9)	6 (2.5)	11 (5.6)	
	Cannot do it	59 (6.4)	33 (6.7)	13 (5.4)	13 (6.6)	
Shopping	No	595 (66.3)	311 (66.0)	166 (70.7)	118 (61.8)	0.250
	Some	99 (11.0)	46 (9.8)	28 (11.9)	25 (13.1)	
	Many	76 (8.5)	39 (8.3)	17 (7.2)	20 (10.5)	
	Cannot do it	127 (14.2)	75 (15.9)	24 (10.2)	28 (14.7)	
Medication	No	750 (80.7)	396 (80.5)	200 (83.7)	154 (77.8)	0.700
	Some	83 (8.9)	44 (8.9)	16 (6.7)	23 (11.6)	
	Many	37 (4.0)	19 (3.9)	9 (3.8)	9 (4.5)	
	Cannot do it	59 (6.4)	33 (6.7)	14 (5.9)	12 (6.1)	
Light housework	No	479 (56.5)	250 (56.6)	145 (63.0)	84 (47.7)	0.098
	Some	146 (17.2)	76 (17.2)	32 (13.9)	38 (21.6)	
	A lot	88 (10.4)	43 (9.7)	24 (10.4)	21 (11.9)	
	Can’t do it	135 (15.9)	73 (16.5)	29 (12.6)	33 (18.8)	
Hard housework	No	265 (30.8)	155 (34.8) ^c^	78 (32.5) ^c^	32 (17.8)	**0.004**
	Some	154 (17.9)	73 (16.4)	43 (18.5)	38 (21.1)	
	Many	155 (18.0)	75 (16.8)	41 (17.6)	39 (21.7)	
	Cannot do it	285 (33.2)	143 (32.1)	71 (30.5)	71 (39.4)	
Money	No	729 (79.8)	377 (78.1)	197 (83.5)	155 (79.5)	0.565
	Some	64 (7.0)	39 (8.1)	12 (5.1)	13 (6.7)	
	Many	42 (4.6)	22 (4.6)	12 (5.1)	8 (4.1)	
	Cannot do it	79 (8.6)	45 (9.3)	15 (6.4)	19 (9.7)	

Resp. Dis., respiratory disease; COPD, chronic obstructive pulmonary disease; ACO, asthma–COPD overlap. No, if they have no limitations in performing the activity; Some, if they have some difficulties, Many, if they have many difficulties; Cannot do it, if they cannot perform the activity without assistance. Chi-square test. Statistical significance: ***p* < 0.05**. Superscripts ^a,b,c^ indicate significant differences in pairwise comparisons after Bonferroni correction. ^a^ Significant differences with COPD; ^b^ significant differences with asthma; ^c^ significant differences with ACO.

## Data Availability

Publicly available datasets were analyzed in this study. These data can be found here: www.ine.es.
